# Burnout syndrome among brazilian medical students under different educational models

**DOI:** 10.1192/j.eurpsy.2021.987

**Published:** 2021-08-13

**Authors:** T. Prata, D. Calcides, E. Vasconcelhos, A. Carvalho, E. De Melo, E. Costa

**Affiliations:** 1 Medicine Department, Federal University of Sergipe, Aracaju, Brazil; 2 University Hospital Of The Federal University Of Sergipe, Federal University of Sergipe, Aracaju, Brazil; 3 Medicine Department Lagarto, Federal University of Sergipe, Lagarto, Brazil; 4 Pharmacy Department Lagarto, Federal University of Sergipe, Lagarto, Brazil

**Keywords:** Burnout Syndrome, Medical Students, mental disorder, Medical Education

## Abstract

**Introduction:**

Medical students are exposed to many stressors which may contribute to the onset of Burnout Syndrome (BS). It consists of a triad of emotional exhaustion, cynicism and low professional efficacy. As a result, BS may reduce academic performance, quality of life and damage future professional life.

**Objectives:**

Estimate the prevalence and recognize associated factors of BS among medical students from two different medical schools form the same Brazilian Public University with different teaching models: School 1, with a traditional model, and School 2, with Problem-Based Learning model.

**Methods:**

A cross-sectional study was performed with randomly selected students between April and June 2019. A structured questionnaire on socio-demographic characteristics and the educational process in addition to The Maslach Burnout Inventory/Student Survey (MBI-SS) were used. Statistical evaluation of multiple variables was performed through backward stepwise logistic regression analysis.

**Results:**

Study included 213 students, with an average age of 23±3.77, 50,2% were male and 62,5% belong to School 1. Among this sample, 21,6% of the students fit tridimensional criteria for BS. Burnout levels were higher in those people who rarely get emotional support they need in the course (OR=3,98, CI 95%, 1,75-9,06), who considered abandoning the course (OR= 2,88, CI 95% 1,29-6,43) and who consider their academic performance regular or weak (OR= 12,1, CI 95%, 4-36,5).

**Conclusions:**

Results suggest a high prevalence of BS with factors associated with the psychosocial and educational sphere of medical students. In our research, the teaching model was not a factor associated with BS.

